# Vitamin A Status of Women and Children in Yaoundé and Douala, Cameroon, is Unchanged One Year after Initiation of a National Vitamin A Oil Fortification Program

**DOI:** 10.3390/nu9050522

**Published:** 2017-05-20

**Authors:** Reina Engle-Stone, Martin Nankap, Alex Ndjebayi, Marie-Madeleine Gimou, Avital Friedman, Marjorie J. Haskell, Ann Tarini, Kenneth H. Brown

**Affiliations:** 1 Department of Nutrition, University of California, One Shields Ave, Davis, CA 95616, USA; mjhaskell@ucdavis.edu (M.J.H.); khbrown@ucdavis.edu (K.H.B.); 2 Helen Keller International, Yaoundé, Cameroon; nankapm@gmail.com (M.N.); andjebayi@hki.org (A.N.); 3 Centre Pasteur of Cameroon, Yaoundé, Cameroon; gimoumm@gmail.com; 4 Helen Keller International, New York, NY 10010, USA; afriedman@hki.org; 5 Independent Consultant, Laval, QC H7G 3Z5, Canada; tariniann@gmail.com; 6 Bill & Melinda Gates Foundation, PO Box 23350, Seattle, WA 98102, USA

**Keywords:** vitamin A, food fortification, cooking oil, breast milk, retinol-binding protein

## Abstract

Vitamin A (VA) fortification of cooking oil is considered a cost-effective strategy for increasing VA status, but few large-scale programs have been evaluated. We conducted representative surveys in Yaoundé and Douala, Cameroon, 2 years before and 1 year after the introduction of a mandatory national program to fortify cooking oil with VA. In each survey, 10 different households were selected within each of the same 30 clusters (*n* = ~300). Malaria infection and plasma indicators of inflammation and VA (retinol-binding protein, pRBP) status were assessed among women aged 15–49 years and children aged 12–59 months, and casual breast milk samples were collected for VA and fat measurements. Refined oil intake was measured by a food frequency questionnaire, and VA was measured in household oil samples post-fortification. Pre-fortification, low inflammation-adjusted pRBP was common among children (33% <0.83 µmol/L), but not women (2% <0.78 µmol/L). Refined cooking oil was consumed by >80% of participants in the past week. Post-fortification, only 44% of oil samples were fortified, but fortified samples contained VA concentrations close to the target values. Controlling for age, inflammation, and other covariates, there was no difference in the mean pRBP, mean breast milk VA, prevalence of low pRBP, or prevalence of low milk VA between the pre- and post-fortification surveys. The frequency of refined oil intake was not associated with VA status indicators post-fortification. In sum, after a year of cooking oil fortification with VA, we did not detect evidence of increased plasma RBP or milk VA among urban women and preschool children, possibly because less than half of the refined oil was fortified. The enforcement of norms should be strengthened, and the program should be evaluated in other regions where the prevalence of VA deficiency was greater pre-fortification.

## 1. Introduction

Vitamin A deficiency (VAD) increases the risk and severity of infections, and was estimated to contribute to tens of thousands of deaths from diarrhea and measles among preschool children worldwide in 2013 [1]. Among many interventions available to increase vitamin A intake and thus minimize the consequences of VAD, the large-scale fortification of staple foods has been highlighted as a cost-effective alternative [2]. In efficacy trials, the consumption of fortified staple foods increased vitamin A status, as measured by plasma concentrations of retinol, among preschool children in Guatemala consuming fortified sugar [3] and children in Indonesia consuming fortified monosodium glutamate (MSG) [4]. The breast milk vitamin A concentration may also be used as an indicator of the response to VA fortification, with the advantage that it does not appear to be subject to homeostatic regulation and thus could potentially respond even when VA stores are adequate [5]; this is in contrast to plasma retinol or retinol-binding protein, which may not change measurably when VA deficiency is mild or when VA stores are adequate. Increases in breast milk retinol were observed following sugar fortification in Guatemala [6] and oil fortification in Indonesia [7]. Although the potential efficacy of large-scale VA fortification is clear, numerous factors may limit the program effectiveness and public health impact, including program compliance by industries, fortificant stability, consumer preferences, and biological factors such as infections, which can lead to increased losses of VA. Despite the introduction of VA fortification programs in dozens of countries [8], few data are available on the effectiveness of these programs for improving the micronutrient status of women and children. Such data are necessary to improve program implementation and confirm changes in the VA status and related outcomes.

VAD has been identified as a public health problem in Cameroon for decades [9,10,11]. In 2009, we conducted a national survey to establish the baseline prevalence of VAD, prior to large-scale fortification with VA, and collected information on dietary patterns to inform the development of the program [12]. In August, 2011, the Government of Cameroon launched a mandatory program to fortify refined vegetable oil with vitamin A. One year later, we conducted a regional survey to evaluate the interim impact of the fortification program in two urban areas (Yaoundé and Douala). In this analysis, we aimed to: (1) determine the availability of adequately fortified oil in households and markets, one year after launching the VA fortification program; (2) evaluate the change in the indicators of VA status from 2009 to 2012 (i.e., two years before, and one year after the introduction of fortified products); and (3) assess whether the consumption of fortified oil was related to VA status.

## 2. Materials and Methods

### 2.1. Study Design and Sampling

This analysis uses data from two multi-stage, cross-sectional, cluster surveys (2009 and 2012). The surveys were conducted in the same season and employed identical methods for sampling and participant recruitment. The baseline survey was conducted in September–December 2009, and was representative nationally and at the level of each of the three survey strata: the North (North, Far North, and Adamaoua administrative regions); the South (all seven remaining regions, with the exception of Yaoundé and Douala); and Yaoundé and Douala (the two major urban areas, comprising ~20% of total population) [12]. Ninety clusters (30 per stratum) were selected using the 2005 census database (the most recent available at the time of the survey) and proportionate to population size sampling. The interim evaluation survey was conducted in October–November 2012, in the same 30 clusters in Yaoundé/Douala (15 per city) that were sampled in the 2009 survey.

The two surveys used identical methods to sample households within each cluster, but different individuals participated in each survey. Sampling within each cluster was conducted as follows: first, a random start point was identified by locating the approximate geographic center of the cluster, walking a straight line from the cluster center to the cluster perimeter in a randomly-selected direction, assigning a number to all households encountered along this line, and randomly selecting a household to begin sampling. Additional households were identified by the systematic sampling of adjacent households.

### 2.2. Sample Size

For both surveys, the target sample size was 10 households (i.e., 10 women and 10 children) per cluster, for a total of 300 households for Yaoundé and Douala combined. Data collection took place two days after sampling and recruitment; thus, in anticipation of an attrition rate of 15–30%, 12–15 households per cluster were selected to ensure that the target sample size for each cluster could be met on the day of data collection. In addition, to collect a total of five breast milk samples per cluster, or 150 per stratum, we recruited an additional two to three lactating women per cluster to provide breast milk samples only [13]; these women did not participate in other data collection activities, such as interviews or blood sampling. 

### 2.3. Participant Eligibility and Consent

Households were eligible to participate if there was at least one child of 12–59 months of age and one woman of reproductive age (15–49 years) who was the child’s primary caregiver. Children and women were eligible to participate if they had lived in the household for at least one month and did not have self-reported “severe fever”, diarrhea with dehydration, or another severe illness at recruitment or between recruitment and data collection (i.e., during the 72 h prior to data collection). If multiple eligible children were present in the household, one child was chosen at random to participate (by drawing from a hat). The index caregiver was selected as the primary caregiver of the eligible child. Caregivers were eligible to provide a breast milk sample if the breastfeeding child was at least one month of age, regardless of whether the lactating woman or breastfeeding child was selected to participate in the full survey.

Women provided informed oral consent for themselves and the child to participate, with permission from the head of the household where appropriate. The surveys were conducted in accordance with the Declaration of Helsinki. The 2009 survey was approved by the National Ethics Committee of Cameroon (Authorization No. 047/CNE/DNM/O9) and the Institutional Review Board of the University of California, Davis (Protocol #200917294). In 2012, the National Ethics Committee had suspended activity during a period of reorganization; thus, approval was obtained from the Cameroon Ministry of Public Health and the Institutional Review Board of the University of California, Davis (Protocol # 364876).

### 2.4. Data Collection

#### 2.4.1. Household Characteristics and Fortified/Fortifiable Food Consumption

Bilingual (French and English) interviewers administered questionnaires to collect information on household demographic and socio-economic characteristics, including information on the household size and composition, educational level and occupation of the caregiver and head of the household, housing material, sources of drinking water and energy for light and cooking, and household possessions.

Information on the consumption of refined cooking oil was collected using a modified version of the Fortification Rapid Assessment Tool (FRAT), which was developed to quickly collect information on the consumption of fortified (or potentially fortifiable) foods [14]. We retained the food frequency aspect of the FRAT questionnaire, but did not retain the use of a partial 24 h dietary recall to estimate the quantity of fortified foods consumed. In the present study, the interviewers administered a food frequency questionnaire (FFQ) which inquired about the consumption of refined oil in different preparations (e.g., oil in fried foods, oil in sauces, etc.). Respondents were asked how many days during the previous seven days they had consumed each food, and the number of times that they consumed the food on the previous day on which the food was consumed. To discriminate between refined (fortifiable) oil and unrefined oil (e.g., red palm oil), data were collected separately for each type of oil that the respondent consumed in the previous seven days. Women provided responses for themselves and for the child.

In 2009 only, 24 h dietary recall interviews with replicates in a ~10% subset (two non-consecutive days of data) were conducted to quantify the total nutrient intakes of women and children. Details of the methods of data collection and analysis are available elsewhere [12,15].

#### 2.4.2. Blood Samples

Biological sample collections took place at a central location within each cluster. Containers for blood collection and storage were covered in foil to prevent exposure to light. Venous blood (5–7 mL) was collected by antecubital (or, for some children, metacarpal) venipuncture into tubes containing lithium heparin (Sarstedt, Nümbrecht, Germany). Blood samples were placed in a cooler with ice packs for <2 h until centrifugation to separate plasma (10 min at 2500× *g*). Plasma was aliquoted within an opaque, portable “hood” to minimize exposure to light and dust during sample handling, and storage vials were covered with aluminum foil.

In 2012 only, an additional 1–2 mL of blood was collected into tubes containing EDTA, for the measurement of hemoglobin and malaria infection in whole blood. For both surveys, hemoglobin was measured in venous blood using a portable photometer (Hemocue, Angelholm, Sweden); for a small number of children for whom sufficient venous blood could not be obtained for hemoglobin measurement, hemoglobin was measured in a capillary sample following a fingerprick. In 2009, current or recent malaria infection was assessed by measuring plasma histidine-rich protein (HRP-2) concentrations using a commercial CELISA kit (Cellabs, Sydney, Australia) [16,17]. In 2012, malaria was assessed in whole blood using a rapid diagnostic test (Malaria Ag Pf/Pan, SD Bioline, Standard Diagnostics, Gyeonggi-do, Korea). Individuals with positive rapid diagnostic test results were treated and referred to the nearest health clinic.

#### 2.4.3. Breast Milk

In both surveys, breast milk samples were collected according to the casual sampling method [13,18]. The mother was asked to feed her child from the fuller breast. After exactly 30 s, the mother transferred the infant to the other breast, and manually expressed 5–10 mL of milk from the first breast into a plastic container covered in aluminum foil. The time of collection and time of the previous feed (from the breast from which milk was collected) were recorded.

The milk fat concentration was measured in triplicate in the field using the creamatocrit method [19]. After swirling the milk to ensure a homogenous distribution of fat, the milk samples were drawn into nonheparinized glass microhematocrit tubes and centrifuged for 15 min at 1500× *g*. The length of the lipid (‘cream’) layer and the total milk column were measured in duplicate using calipers to the nearest 0.1 mm. The median coefficients of variation (CVs) were 3.8% in 2009 and 3.1% in 2012 (mean = 4.9% for both). After removing an aliquot for milk fat analysis, the remaining milk sample was remixed and aliquoted into storage vials wrapped in foil.

#### 2.4.4. Fortified Foods

During the interview, respondents were asked whether they had any refined oil in their home and, if so, whether they were willing to provide a sample for micronutrient analysis. Samples of ~10 g of oil were collected into sterile, plastic containers covered in foil for protection from light. The VA content of the oil samples was measured on the day of collection using a portable photometric instrument (iCheck Chroma, Bioanalyt, GmbH), according to the manufacturer’s instructions [20]. This version of the instrument is intended for measurement of the common types of oil consumed in Yaoundé/Douala (palm and groundnut), but not cottonseed or soybean oil, so five household oil samples reported to be cottonseed or soybean oil were excluded from the analyses.

#### 2.4.5. Sample Storage and Shipping

Aliquots of plasma, breast milk, and cooking oil were stored in a cooler with ice packs until the end of the day, when they were transferred to a freezer (with a backup generator) for storage at ≤−20 °C. Samples were shipped on dry ice to Germany for an analysis of the plasma proteins (2009 and 2012) at the VitA-Iron lab, and to the United States for an analysis of the breast milk retinol (2009 and 2012) and plasma HRP2 (2009 only) at UC Davis.

#### 2.4.6. Laboratory Analyses

Plasma indicators of inflammation (C-reactive protein, CRP, and α_1_-acid glycoprotein, AGP), and vitamin A status (retinol-binding protein, RBP) were measured using ELISA [21]. In 2009, the interassay CVs were: RBP, 2.7%; CRP, 6.5%; and AGP, 3.5%. In 2012, the CVs of a plasma pool control sample on 11 plates were: RBP, 3.3%; CRP, 4.5%; and AGP, 5.5%.

Plasma HRP2 was detected using a commercial CELISA kit (Cellabs Pty, Ltd., Brookvale, Australia), following the manufacturer’s instructions, with positive and negative controls provided by the manufacturer. Samples with an absorbance value greater than the optical density of the negative control +0.05 U were considered positive for the *P. falciparum* antigen.

Breast milk retinol concentrations were measured under dim or yellow light by a reverse-phase HPLC system (Class VP; Shimadzu) [13,22]. After thawing at room temperature, milk samples were gently vortexed to homogenize them and were then saponified for 1 h at 60 °C in ethanolic KOH with pyrogallol. Retinal (O-ethyl) oxime was added as an internal standard prior to extraction with hexane [23]. Samples were then dried under nitrogen, reconstituted in methanol, and injected onto a 3-μm C18 column using a mobile phase of 68% acetonitrile, 20% isopropanol, and 12% methanol by volume. Retinol was detected at 325 nm with a photo diode-array detector. Infant formula from the National Institute of Standards and Technology (NIST) was analyzed along with each batch of breast milk samples, according to the same procedures (NIST Standard Reference Material 1849, three to four NIST aliquots per batch). Retinol concentrations of the unknowns were calculated by comparing the ratio of retinol to retinal oxime in the NIST formula with that in the unknowns. The measured retinol concentration of the NIST formula was verified using NIST serum (SRM 968e). The within-day and between-day CVs of the NIST controls over the course of analysis were 3.5% and 8.9%, respectively, in 2009, and 3.6% and 13.4%, respectively, in 2012 (SAS proc varcomp).

### 2.5. Statistical Analysis

Data were analyzed using SAS 9.4 (SAS Institute Inc., Cary, NC, USA), with SAS survey analysis procedures. Weighting factors were applied to account for the respective population sizes of Yaoundé and Douala, and to adjust for the non-response within each cluster. For each survey, variables relating to socio-economic status were combined using factor analysis to create a score for socio-economic status. For the present analyses, the scores for the 2009 national survey were recalculated to represent Yaoundé/Douala only, for consistency with the later survey.

We calculated the frequency of the consumption of fortified oil in the past seven days by multiplying the number of days on which the food was consumed by the number of times per day that the food was consumed (on the most recent day on which the food was consumed) [12]. Red palm oil and groundnut oil were excluded from the calculations of refined oil intake.

Details of the analysis of 24 h recall data have been reported elsewhere [15]. Following the calculation of total nutrient intakes, the NCI method was used to estimate the usual intake distributions [24]. We then simulated the effects of the fortification levels measured in this survey on dietary adequacy, as described previously [15].

To report the mean RBP concentrations and prevalence of deficiency, RBP concentrations were adjusted for inflammation using regression analysis, employing a method adapted from Larson et al. [25]. Separate linear regression models for women and children were developed to describe the relationship with CRP and AGP, including interactions or quadratic terms that were significant. These equations were then used to adjust individual values to concentrations equivalent to those in the absence of inflammation, defined as CRP and AGP concentrations of individuals at the 10th percentile of a group with CRP < 5 mg/L and AGP < 1 g/L. The reference CRP and AGP values derived from this dataset were 0.12 and 0.57 for children and 0.16 and 0.47 for women, respectively. However, as described below, differences in biomarker concentrations between the two surveys were examined using the unadjusted concentrations as the outcome variables, and controlling for CRP and AGP concentrations (as continuous variables).

We used previously-derived population-specific cutoffs to define VAD (inflammation-adjusted pRBP < 0.78 µmol/L for women and <0.83 µmol/L for children) and low VA status (pRBP < 1.17 µmol/L for women) [11].

The change in micronutrient status and other indicators over time was examined using SAS survey regression procedures (proc surveyreg), with a binary variable representing pre- or post-fortification samples. Continuous outcome variables were examined for adherence to a normal distribution by the examination of histograms and Shapiro Wilke’s “W” [26], and were transformed where necessary to achieve a normal distribution (W ≥ 0.97).

To control for potential confounding in the relationship between refined oil intake and VA status, we used logistic regression to create propensity scores for the frequent consumption of refined oil [27]. We defined frequent consumption as ≥14 times/week, approximately the 75th percentile of consumption. Because “brand-name” cooking oil was more likely to be fortified than “bulk” oil, we also developed a separate score for the consumption of “brand-name” oil ≥ 7 times/week. Predictor variables included variables related to socio-economic status, including the type and location of residence; housing materials and the type of toilet; sources of lighting, water, and energy for cooking; occupation and employment status of the caregiver and head of the household; and caregiver education. The calculated propensity score for the total refined oil consumption was correlated with the frequency of refined oil intake (women: r_s_ = 0.31; children: r_s_ = 0.29, *P* < 0.0001 for both), and the probability score for branded oil intake was correlated with branded oil intake (women: r_s_ = 0.31, *P* < 0.0001; children: r_s_ = 0.35, *P* < 0.0001), but not with RBP among women or children (*P* > 0.14). The probability score for the total, but not branded-only, refined oil intake was marginally correlated with inflammation-adjusted RBP among children (r_s_ = 0.08, *P* = 0.051), but not women (*P* = 0.86).

For the adjusted analyses of difference in vitamin A biomarkers between surveys, variables were considered as potential covariates if they were correlated with either the outcome or with the refined oil consumption. Selected covariates were also included for theoretical reasons. Potential covariates included: age; residence in Yaoundé or Douala; household socioeconomic status (continuous score); CRP and AGP (both continuous); current or recent malaria; type of toilet used by the household (proxy for household sanitation and pathogen exposure); propensity to consume refined oil ≥14 times/week or branded refined oil ≥7 times/week; reported receipt of a vitamin A supplement in the previous six months, breastfeeding status, height-for-age Z-score, and weight-for-age Z-score (children); pregnancy or lactation, BMI (women); and receipt of postpartum vitamin A supplement, age of the breastfeeding infant, and milk fat content (breast milk vitamin A).

Square terms were evaluated where the relationship between the covariate and outcome variable did not appear linear, and selected interactions with CRP, AGP, and the child’s age were included. All possible covariates and selected interaction terms were then added to the regression model, and covariates were sequentially removed (beginning with interactions) if they were not significantly associated with the outcome (*P* > 0.05; *P* > 0.1 for interactions). If the removal of a covariate changed the regression coefficient of the “survey year” variable by more than 20% or caused the P value of the outcome to cross the threshold for statistical significance, the covariate was retained in the model. Age was retained in all models. Regression diagnostics, including measures of collinearity, normality of residuals, and leverage, were examined for “full models” (all covariates) and final models.

Finally, we conducted several plausibility analyses to assess whether any observed change over time was related to the consumption of fortified foods. First, we examined the relationships between micronutrient status indicators and the frequency of refined oil consumption post-fortification using Spearman correlations (using the SAS correlations procedure; Spearman, rather than Pearson, correlations were chosen because the frequency of oil intake was not normally distributed). Second, because we observed previously that the frequency of consumption of some foods was related to micronutrient status (likely because of the associations of each with underlying factors like socioeconomic status [12]), to better assess whether the relationship between fortified food intake and micronutrient status differed pre- and post-fortification, we modeled this as the interaction between the survey year and frequency of consumption of fortified foods in regression models predicting concentrations of each VA biomarker. However, this was an exploratory analysis, because we did not base the sample size on that required to detect interactions.

## 3. Results

### 3.1. Participant Characteristics

The characteristics of the participants in the 2009 survey, including micronutrient status and dietary intake, have been reported in detail elsewhere [10,12]. For the 2012 survey, 704 households were approached to assess eligibility. Of these, eligibility could not be assessed for 82 households, primarily due to the absence on the day of recruitment or refusal of eligibility assessment, and 191 households were not eligible for the full study (of which 149 were not eligible because the household did not include a child of 12–59 months of age). Of the 428 households that were eligible for the full survey, 425 consented to participate, and 333 participated in some aspect of data collection. There were no differences in housing materials (floor and walls) and the language of interview (English or French) among households that were eligible, households that were not eligible, and households whose eligibility could not be assessed; the same characteristics did not differ among households that consented and did or did not participate (*P* > 0.25 for all). Reasons for non-participation were not recorded for individual HH, but included: the woman or child was ill, the woman was too busy, the woman or child had traveled outside the cluster, and the head of the household later refused.

Of the 191 households not eligible for the full study, 94 included a woman who was eligible to provide breast milk, 53 did not include an eligible lactating woman, and for 44 households, the eligibility to provide breast milk only could not be assessed. Of the 94 women eligible to provide breast milk only, 93 consented to provide breast milk, and milk samples were available for 72 women. There were no differences in the wall or floor material, or language of interview among households that did or did not include a woman eligible to provide breast milk, or whose eligibility to provide breast milk only could not be assessed; the same characteristics did not differ between women who consented to provide breast milk only and did or did not participate (*P* > 0.50 for all).

The characteristics of participants in the pre- and post-fortification surveys were generally similar (Table 1). Women in the post-fortification survey were older (29.1 vs. 27.1 year, *P =* 0.002) and had higher mean CRP concentrations (3.5 vs. 2.7 mg/L, *P =* 0.0002), but mean AGP concentrations, the prevalence of inflammation, malaria, the proportion of pregnant or lactating women, postpartum vitamin A supplement receipt, and milk fat concentration did not differ. Children in the post-fortification survey were also slightly older (32.8 vs. 30.3 months, *P* = 0.039) and less likely to have received a high dose vitamin A supplement in the past six months (50 vs. 76% according to caregiver report, *P* < 0.001) compared to children in the pre-fortification survey. However, child sex, mean CRP and AGP concentrations, the prevalence of inflammation and malaria, and the proportion of children breastfeeding did not differ between participants in the two surveys.

### 3.2. Micronutrient Content of Refined Oil Samples

Among 197 refined oil samples collected from the households, the type of oil was reported to be vegetable oil (nonspecified type) in 60% of samples and palm oil in 35% of samples; cottonseed, soybean, and groundnut oil represented < 2% each. After the exclusion of five cottonseed and soybean oil samples, “brand name” samples accounted for 35% of the samples collected, and 60% were “bulk” samples (5% unknown). Samples of bulk (“*en vrac*”) vegetable oil were generally sold in small secondary containers such as bottles and sachets in open air markets or neighborhood kiosks, and are thought to be mainly refined palm oil. Overall, 44% of samples contained detectable amounts of vitamin A, but “brand name” samples were almost twice as likely as “bulk” samples to contain VA (*P* < 0.001; Table 2). The proportion of samples that were fortified was greater in Yaoundé (54%) compared to Douala, where most imported cooking oil arrives at the port (35%; *P* < 0.05). There was a marginally significant interaction between city and “brand vs. bulk” in predicting the proportion of oil samples that were fortified (*P =* 0.072): over 70% of “brand name” oil samples were fortified in both cities, whereas, among “bulk” oil samples, 41% in Yaoundé, but only 10% in Douala, were fortified.

Among oil samples that contained detectable amounts of vitamin A, the VA concentrations were 11.5 µg RE/g on average, which is very close to the target level of 12 µg RE/g, although the mean VA concentration (among samples with detectable VA) was greater among “brand-name” compared to “bulk” oil samples (12.9 vs. 9.6 µg RE/g, respectively, *P* = 0.002).

### 3.3. Consumption of Fortified Foods and Predicted Impact on Dietary Adequacy

The consumption of refined cooking oil was common among women and children in these urban areas (Table 3). More than 80% of respondents reported consuming refined oil in the past week, with an average frequency of one to two times per day. A greater proportion of respondents reported consuming refined oil in the past week in 2012 compared to 2009, but the frequency of consumption did not differ, except for a decrease among children who consumed oil in the past week (Table 3). Controlling for respondent age did not change these relationships (data not shown).

Pre-fortification, 59% of women and 64% of non-breastfeeding children had a VA intake below the respective estimated average requirement (EAR) values (<500 and <210 µg RAE/d). Simulations using 24-h dietary recall data suggested that fortification with 5.28 mg/kg oil (44% of the target level of 12 mg/kg) would decrease the prevalence of inadequate intakes to 16% among women and 38% among non-breastfeeding children in this region (compared to the predicted prevalence of ~45–50% nationally post-fortification [15]).

### 3.4. Change in Biomarkers of Vitamin A Status

Prior to fortification, the prevalence of VAD (pRBP < 0.83 µmol/L) among children was 30.6%, which is greater than the 20% threshold adopted by WHO to define VAD as a public health problem [28] (Table 4). Among women, the prevalence of deficiency was low (less than 5% <0.78 µmol/L), but 10.3% had plasma RBP concentrations consistent with low VA stores (<1.17 µmol/L; Table 4).

**Figure 1:**
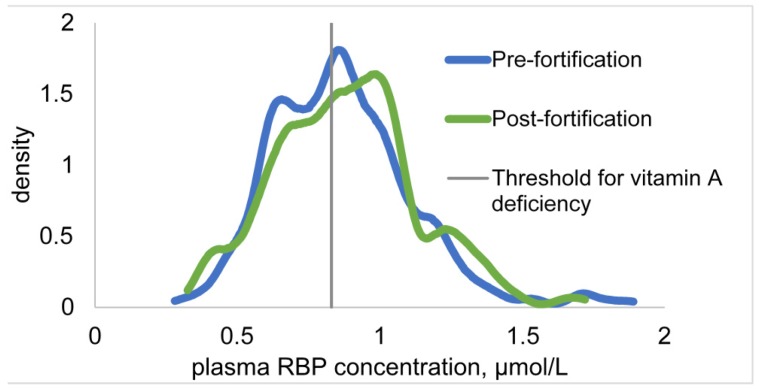
Kernel density distributions of plasma retinol-binding protein (RBP) concentrations (unadjusted for inflammation) among children 12–59 months of age in Yaoundé and Douala, Cameroon, two years before and one year after the introduction of vitamin A-fortified cooking oil through a mandatory national program. Plasma RBP is compared to the cutoff for vitamin A deficiency (0.83 µmol/L, equivalent to 0.70 µmol/L plasma retinol).

The distributions of plasma RBP concentrations pre-fortification and post-fortification were similar (Figure 1). There were no differences between surveys in mean plasma RBP concentrations, or the prevalence of low RBP values among children or women, with or without controlling for covariates. The covariates that remained significant in the models of plasma RBP concentration among women were age, CRP, AGP, BMI, type of household toilet, stratum (Yaoundé vs. Douala), and an interaction between CRP and AGP. For children, the covariates retained for comparisons of the mean RBP concentration were age, CRP, AGP, breastfeeding status, household socioeconomic status, and an interaction between age and breastfeeding status. For the prevalence of low RBP concentration among children, the covariates were age, CRP, AGP, the interaction between CRP and AGP, weight-for-age Z-score, vitamin A supplement (VAS) receipt in the past six months, and propensity to consume fortified oil.

**Figure 2:**
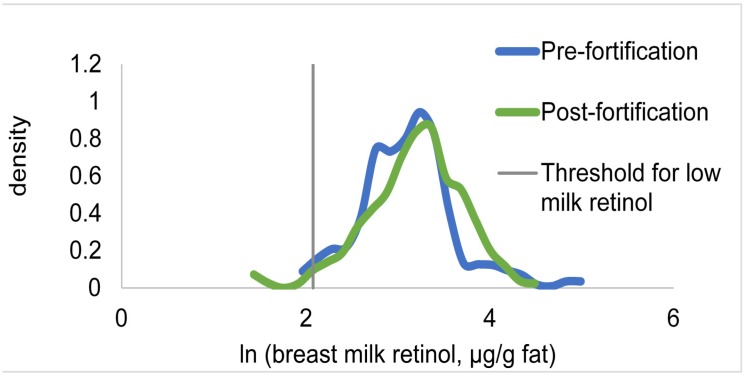
Kernel density distributions of breast milk vitamin A concentration (expressed as µg/g fat and subjected to natural logarithm transformation) among a representative sample of breastfeeding women in Yaoundé and Douala, Cameroon, two years before and one year after the introduction of vitamin A-fortified cooking oil through a mandatory national program. Breast milk vitamin A concentration is compared to the cutoff for low milk vitamin A concentration, 8 µg/g fat.

Prior to fortification, breast milk vitamin A concentrations were adequate: < 3% of women had BMVA < 8 µg/g fat (Table 4). Although the distribution of BMVA appeared to shift slightly to the right (Figure 2), the mean BMVA concentrations did not differ between 2009 and 2012 (β ± SE = 0.12 ± 0.08, *P* = 0.15), controlling for age of the breastfeeding child, milk fat concentration, and a square term for milk fat.

### 3.5. Plausibility Analyses

Post-fortification, there were no relationships between the frequency of refined oil intake (with or without restriction to intake of “branded” oil) and RBP concentrations among women or children, with or without the adjustment of RBP for inflammation. In addition, the frequency of refined oil intake in the past week was not associated with breast milk VA concentrations.

There was a marginally significant interaction (*P* = 0.052) between the survey year and frequency of refined oil intake on the proportion of women with RBP < 0.78 µmol/L. However, the prevalence of VAD among women was <4% in both years, with or without adjustment for inflammation. The interaction between the survey year and frequency of cooking oil consumption was not statistically significant for plasma RBP concentrations among women or children, or for the prevalence of low RBP concentrations among children.

## 4. Discussion

We evaluated the availability and consumption of adequately-fortified refined oil and biomarkers of VA status in a representative sample of women and young children in two large cities in Cameroon, one year after the introduction of VA-fortified cooking oil mandated by a national program. We observed that the consumption of refined oil was frequent, but only 44% of oil samples were fortified, indicating the need to strengthen efforts to monitor the quality of fortified products and enforce the norms adopted by the government.

Simulations using dietary intake data from 24-h recalls (collected pre-fortification) suggested that the fortification of refined oil at 44% of the target fortification level would reduce the prevalence of inadequate intakes. However, we found no evidence of a change in mean plasma retinol-binding protein or breast milk vitamin A concentrations, or the prevalence of low values, between surveys conducted two years before and one year after program implementation. This finding does not exclude the potential for benefit among subgroups, such as those with low vitamin A status prior to fortification, which may not have been detectable with this sample size. However, the absence of a large effect of the program is not surprising as less than half of the oil collected from the households was fortified.

The results also do not exclude potential benefits in other regions, but the resources did not permit the collection of data elsewhere in the country. Prior to fortification, the prevalence of VAD was higher among both women and children in the northern regions, suggesting that the response to fortification would be greatest in this area; however, the consumption of locally-produced groundnut oil (which is not included in the current VA fortification program) was common in the northern regions of Cameroon [12], which may limit the overall impact of refined oil fortification with VA.

Among children in Yaoundé and Douala, the median consumption of refined oil, among consumers, was 13 g/day prior to fortification; the corresponding value for women was 26 g/day. At the target fortification level (12 µg/g), this would deliver 156 µg retinol/d to children who consume refined oil, or 74% of the EAR; for women, the additional VA consumed, on average, would be 312 µg retinol/day, or 62% of the EAR. However, assuming an average fortification level of 5.3 µg/g (the average among fortified and unfortified samples), the amount of vitamin A delivered each day would be reduced to 69 µg/day for children and 134 µg/day for women, and any losses from cooking would further decrease the total amount of VA delivered. Almost one third of children had low RBP concentrations at the baseline, so plasma RBP concentrations would be expected to respond to an increased VA intake, as observed elsewhere [3,7,29]. Thus, fortification levels below the target and an inadequate sample size may explain the lack of a detectable effect on plasma RBP concentrations among children.

More children reportedly received periodic high-dose vitamin A supplements in the past six months in 2009 (76%) compared to 2012 (50%), which could result in relatively greater RBP concentrations in the pre-fortification survey. The VAS distribution campaign was conducted ~3 months in advance of the survey in Yaoundé and Douala in 2009 and ~4 months in advance of the survey in 2012, so receipt of VAS would likely have a minimal impact on the indicator of vitamin A status by this time. An indicator variable for reported VAS receipt was included in the models; this variable was a significant predictor of low RBP, but not RBP concentration. Thus, it is unlikely that the timing and coverage of the VAS campaigns influenced the results, but the possibility cannot be completely ruled out.

Among women, plasma RBP concentrations were not low at the baseline, so this indicator would not be expected to respond to increased VA intake among women. Although breast milk VA concentrations expressed as µmol/L appeared to increase, the increase was not statistically significant after controlling for milk fat concentration, which is necessary because variation in milk fat can influence milk VA concentrations. The lack of effect on breast milk VA, which can potentially respond to increased VA intake, even when the status is adequate, is unexpected, but may also reflect low amounts of additional VA intake and the sample size. Assuming a median oil consumption of 26 g/day (among consumers) and 44% target fortification levels, the program would deliver, on average, ~137 µg retinol/day, prior to cooking. Plasma and breast milk retinol concentrations increased among Indonesian women who consumed similar amounts of fortified oil, but average amounts of VA in oil at the household level (8.6 µg/g) and the prevalence of deficiency at the baseline were greater [7].

Significant investments are required for the design and implementation of large-scale fortification programs. These results emphasize the need for continued monitoring following program launch to ensure that the desired impacts are achieved, as highlighted for other settings [30]. Overall, 56% of the oil samples collected from households in the post-fortification survey were not fortified, and, notably, 74% of “bulk” oil samples were not fortified. This latter oil is sold in unlabeled or in secondary containers (e.g., in water bottles) and is thought to represent both imported and locally produced palm oil. The quality of vegetable oil after refining [31] and exposure to light and heat while displayed for sale in open markets may also contribute to the degradation of VA in samples. However, the samples with detectable VA contained mean VA concentrations close to the target levels, suggesting that the primary explanation is that the oil was never fortified, rather than varying degrees of VA loss following adequate fortification. It is possible that some refined oil that was produced prior to the introduction of fortified products was still available on the market one year later. Both domestically-refined and imported oil are subject to mandatory fortification, but monitoring activities are currently limited. The institution of a system for the regular monitoring of both domestic and imported products is necessary to increase the potential of the program to address VAD.

## 5. Conclusions

In conclusion, refined oil is widely consumed by women and children in urban Cameroon, but less than half of the oil was fortified one year following the launch of the program. We found no evidence of change in VA status post-fortification, although our small sample size means that we cannot exclude the benefit in some subgroups, or in regions that were not studied, where the prevalence of VAD was greatest prior to fortification. Support for program monitoring and facilitating compliance is urgently needed to improve the ability of the program to increase VA intakes and status among young children.

## Figures and Tables

**Table 1:** Characteristics of women and children in Yaoundé and Douala, Cameroon who participated in the baseline and post-fortification surveys.

		Pre-Fortification (2009)		Post-Fortification (2012)		*P* Value
		*n*	Mean ± SE or %	*n*	Mean ± SE or %	
Women						
	Age, year	279	27.1 ± 0.4	302	29.1 ± 0.4	0.002
	Pregnant, %	214	11	293	16	0.07
	Lactating, %	228	26	302	28	0.71
	Received postpartum VAS ^1^ (among lactating women) ^2^, %	106	43	131	47	0.54
	Milk fat content ^2^, g/L	130	47.1 ± 2.4	151	51.7 ± 3.5	0.075
	CRP, mg/L	273	2.67 ± 0.22	305	3.52 ± 0.37	0.0002
	AGP, g/L	273	0.73 ± 0.01	305	0.72 ± 0.01	0.070
	Inflammation, %	273	18	305	22	0.19
	Malaria, %	261	7	299	5	0.39
Children						
	Age, year	272	30.3 ± 1.0	303	32.9 ± 0.8	0.036
	Male, %	288	49	308	50	0.86
	Stunted (HAZ < −2), %	255	13.0	300	15.6	0.29
	Breastfeeding, %	239	5	281	4	0.69
	Received VAS in past 6 month, %	233	76	289	51	0.002
	CRP, mg/L	254	4.20 ± 0.33	297	4.49 ± 0.45	0.58
	AGP, g/L	254	0.90 ± 0.02	297	0.97 ± 0.02	0.12
	Inflammation, %	254	38	297	46	0.10
	Malaria, %	234	13	294	8	0.088

^1^ AGP, α_1_-acid glycoprotein; CRP, C-reactive protein; HAZ, height-for-age Z-score; VAS, vitamin A supplement. ^ 2^ Includes women who provided breast milk only.

**Table 2:** Vitamin A content of refined oil samples collected from households in Yaoundé and Douala ^1^.

		Yaoundé		Douala		Total	
		*n*	Mean (95% CI)	*n*	Mean (95% CI)	*n*	Mean (95% CI)
Detectable VA, %							
	“Brand name” oil	28	81.4 (65.7–97.1)	39	71.8 (56.4–87.2)	67	75.6 (64.8–86.3)
	“Bulk” oil	62	40.6 (28.4–52.8)	53	10.1 (0.6–19.6)	115	26.1 (18.1–34.1)
	Overall^2^	96	54.0 (44.0–63.9)	96	34.9 (21.5–48.4)	192	44.1 (36.1–52.2)
Average VA concentration, among samples with VA, µg RE/g							
	“Brand name” oil	23	13.8 (11.7–16.0)	26	12.1 (10.7–13.6)	49	12.9 (11.6–14.1)
	“Bulk” oil	25	9.9 (8.4–11.3)	5	8.3 (3.2–13.4)	30	9.6 (8.1–11.0) ^3^
	Overall ^2^	52	11.5 (9.9–13.0)	31	11.5 (10.1–12.9)	83	11.5 (10.5–12.5)

^1^ All oil samples were collected from households. Results exclude 5 samples reported to be cottonseed or soybean oil because the instrument for vitamin A measurement was not validated for use with these oils at the time of the survey. ^2^ Includes 10 oil samples (4 of which contained vitamin A) for which the oil brand was “other” or not known. ^3^ Different from mean VA content of “Brand name” oil samples, *P* = 0.002.

**Table 3:** Consumption of fortified foods and predicted change in the adequacy of micronutrient intake in Yaoundé/Douala following the fortification of refined oil ^1^.

	Women			Children		
	Pre-fortification (2009)	Post-fortification (2012)	*P* value	Pre-fortification (2009)	Post-fortification (2012)	*P* value
N (FFQ)	290	309		290	309	
Refined oil consumption in past week, %	82.4 ± 2.8	93.6 ± 1.4	<0.0001	81.2 ± 3.0	91.7 ± 1.5	0.0002
Frequency of oil consumption in past week, among consumers, times/wk	10.3 ± 0.5	9.2 ± 0.3	0.14	10.7 ± 0.6	9.1 ± 0.5	0.009
Frequency of refined oil consumption, all participants, times/wk	8.5 ± 0.5	8.6 ± 0.4	0.13	8.7 ± 0.6	8.3 ± 0.5	0.59
N (24-h dietary recall)	297	-	-	229	-	-
Mean oil intake in the past day, among consumers, g/day	32 ± 2	-	-	18 ± 2	-	-
Mean usual oil intake (total population) ^2^, g/day	17.2 ± 0.6	-	-	10.1 ± 0.4	-	-
Total usual VA intake ^2^, µg RAE/d	449 ± 20	630 ± 20	-	194 ± 8	285 ± 9	-
Vitamin A intake < EAR ^2^, %	59 ± 15	16 ± 2	-	64 ± 2	38 ± 5	-

^1^ Values are mean ± SE. Simulations assume oil fortification at 44% of the target level (final value: 5.28 mg/kg). ^2^ Calculated using the National Cancer Institute (NCI) method^15^. For children, estimates of dietary adequacy were calculated only among non-breastfeeding children, because breast milk intake was not measured. Prevalence of inadequate intake was calculated using the estimated average requirement (EAR) cut point method. RAE, retinol activity equivalent.

**Table 4:** Indicators of vitamin A status among women and children who participated in the baseline and post-fortification studies, and change from 2009 to 2012 ^1^.

		Pre-Fortification (2009)	Post-Fortification (2012)	*P* ^2^	*P* ^3^
Women					
	N	273	305		
	RBP, µmol/L	1.41 ± 0.02	1.40 ± 0.02	0.70	0.35
	Adjusted^4^ RBP, µmol/L	1.65 ± 3.0	1.66 ± 0.02	0.34	-
	RBP < 0.78 µmol/L, %	2.4 ± 0.9	3.4 ± 1.1	0.54	0.58
	RBP < 1.17 µmol/L, %	22.5 ± 3.1	25.9 ± 2.9	0.42	0.28
	Adjusted ^4^ RBP < 0.78 µmol/L, %	1.5 ± 0.7	1.7 ± 0.7	0.83	-
	Adjusted ^4^ RBP < 1.17 µmol/L, %	10.3 ± 1.9	14.8 ± 1.7	0.80	-
	N	134	154		
	Breast milk vitamin A, µmol/L	3.67 ± 0.21	4.38 ± 0.25	0.011	0.15
	Breast milk vitamin A, µg/g fat	26.1 ± 1.8	27.1 ± 1.2	0.41	-
	Breast milk vitamin A < 1.05 µmol/L, %	7.6 ± 1.8	2.5 ± 1.2	0.050	0.85
	Breast milk vitamin A < 8 µg/g fat	2.5 ± 1.9	2.6 ± 1.5	0.96	-
Children					
	N	254	297		
	RBP, µmol/L	0.87 ± 0.02	0.88 ± 0.02	0.68	0.18 ^5^
	Adjusted ^4^ RBP, µmol/L	0.98 ± 0.02	1.00 ± 0.02	0.49	-
	RBP < 0.83 µmol/L, %	44.2 ± 3.6	41.2 ± 3.2	0.50	0.18 ^6^
	Adjusted ^4^ RBP < 0.83 µmol/L, %	30.6 ± 3.9	26.6 ± 2.2	0.28	-

^1^ Values are mean ± SE. ^2^ Unadjusted, unless otherwise indicated. ^3^ Adjusted for participant characteristics and relevant confounders. ^4^ Values adjusted for inflammation by regression analysis to values equivalent to those at CRP and AGP concentrations of 0.12 mg/L CRP and 0.57 g/L AGP for children and 0.16 mg/L CRP and 0.47 g/L AGP for women (the 10th percentile among individuals with CRP < 5 and AGP < 1). Values for the two surveys were compared by regression analysis, using the unadjusted value as the dependent variable and including CRP, AGP, and their interaction as covariates. ^5^ Including brand-name oil intake, rather than all refined oil, as a potential covariate, *P* = 0.15. ^6^ Including brand-name oil intake, rather than all refined oil, as a potential covariate, *P =* 0.049.
